# The Classical–Quantum Passage: A van der Waals Description

**DOI:** 10.3390/e24020182

**Published:** 2022-01-26

**Authors:** Flavia Pennini, Angel Plastino

**Affiliations:** 1 Departamento de Física, Universidad Católica del Norte, Av. Angamos 0610, Antofagasta 3580000, Chile; fpennini@ucn.cl; 2 Departamento de Física, Facultad de Ingeniería, Universidad Nacional de Mar del Plata (UNMDP), CONICET, Mar del Plata 7600, Argentina; 3 Instituto de Física La Plata–CCT-CONICET, Universidad Nacional de La Plata, C.C. 727, La Plata 1900, Argentina

**Keywords:** noble gases, thermal efficiency, van der Waals gas

## Abstract

We undertake a van der Waals inquiry at very low temperatures so as to find signs of a classical–quantum frontier. We investigate the relation of such signs with the celebrated van der Waals gas–liquid transition. We specialize the discussion with respect to the noble gases. For such purpose, we use rather novel thermal statistical quantifiers such as the disequilibrium, the statistical complexity, and the thermal efficiency. Fruitful insights are thereby gained.

## 1. Introduction

The van der Waals (vdW) fluid is the simplest instance of a system endowed with interacting particles. It is well known that it exhibits a gas–liquid phase transition (GLPT) [1,2,3,4,5,6,7,8,9,10]. We wish to point out here that there exists a second vdW instability, at low temperatures *T*, that could be interpreted as pre-announcing the classical quantum passage (CQP) [11] (see also Ref. [12]).

An important proviso: we definitely not studying quantum phase transitions such as those found in the excellent book [13] or the interesting papers [14,15,16]. We are dealing only with a classical equation and investigating its low *T* validity limits. At low temperatures, we find indeed one such limitation that is interpreted as stated above.

More precisely, we ask how the GLPT and the putative CQP are related. Is there an interplay between them? This effort is devoted to answer these questions, using the noble gases as the target-system and rather novel thermal statistical quantifiers as the appropriate mathematical tools. We will try to set the ensuing discussion into an order–disorder disjunction framework, using the thermal quantifiers referred to above. They are the thermal efficiency η, the statistical complexity *C*, and the disequilibrium *D*. Further, *C* is intimately associated to *D* via the entropy *S* [17]

(1) C=SD.

We point out that it is well known that *C* and *D* are useful quantities for detecting phase transitions [17].

### 1.1. Special Thermal Quantifiers Used Here

Consider some particular probability distributions (PDs). Maximum statistical disorder is associated to a uniform probability distribution (UF) [18]. The more dissimilar the extant PD is from the UF, the more statistically “ordered” this PD is [18]. The dissimilarity we are speaking about is linked to a distance in probability space between our actual PD and UF called the disequilibrium *D* [18]. Note that [18]

(2) 0≤D≤1.

As stated above, the quantifier called statistical complexity *C* is given by [18] C=DS. Such C−form is today routinely employed in dozens of papers [19,20,21,22,23,24,25,26,27]. In addition, we use the standard forms for the Helmholtz’ free energy *F* [1]. With it, we introduce a further thermal quantifier called the thermal efficiency η [17,28] of an arbitrary control parameter *X* of our system

(3) $$\eta =-\frac{1}{k_{B}}\frac{\partial S/\partial X}{\partial F/\partial X},$$

that represents the work needed to change from *X* to X+dX [28].

### 1.2. Goal

In this work, we use the thermal quantifiers *D*, *C*, and η to scrutinize the relations that may exist between the vdW’s (1) liquid–gas transition (GLPT) and (2) putative quantum–classic frontier (CQ). Some rewarding insights will be obtained.

### 1.3. Organization

This paper has the following organization. In Section 2, we introduce preliminary concepts. In Section 3, we present our results and exhaustively discuss the relation between our GLPT and the CQP for five noble gases. Finally, we draw some conclusions in Section 4.

## 2. Basic Relations for Ideal and van der Waals Gases

To undertake the present endeavor, we collect in this section some basic gas relations, as recounted in Refs. [11,12]. We will need expressions for the partition function, the mean energy *U*, the free energy *F*, etc.

The ideal gas is a collection of *N* identical particles contained in a volume *V*. One assumes thermal equilibrium at temperature *T*. The Hamiltonian reads $H_{0}=\sum_{i=1}^{N}\;p_{i}^{2}/2m$. *m* is the mass and $p_{i}$ the momenta, i=1,…,N. One deals with a canonical partition function [1]

(4) $$Q_{N}^{\left(0\right)}\left(V,T\right)=\int d\Omega \;\exp\left(-\beta \;H_{0}\right)=\frac{1}{N!}{\left(\frac{V}{\lambda^{3}}\right)}^{N},$$

where $d\Omega =d^{3N}r\;d^{3N}p/N!h^{3N}$. λ is the average thermal wavelength

(5) $$\lambda ={\left(\frac{2\pi \hbar^{2}}{mk_{B}T}\right)}^{1/2}.$$

The free energy becomes [1]

(6) $$F_{ideal}=-Nk_{B}T\;\left[\ln\left(\frac{v}{\lambda^{3}}\right)+1\right],$$

with v=V/N the specific volume. [1]. Having *F*, one also has all the important thermodynamic quantities, that we enumerate below, and one also deals with the chemical potential [1]

(7) $$\mu_{ideal}=k_{B}T\ln\left(\frac{\lambda^{3}}{v}\right).$$

The classical entropy (Sackur–Tetrode equation) is

(8) $$S_{ideal}=-Nk_{B}\ln\left(\frac{\lambda^{3}}{v}\right)+\frac{5}{2}Nk_{B}=Nk_{B}\ln\left(e^{5/2}\;\left(\frac{v}{\lambda^{3}}\right)\right),$$

One has S>0 for $\lambda^{3}/v\ll e^{5/2}$ [12], which is already telling us just where the classical regime is tenable. S<0 indicates that the classical description is failing [12].

Next, the internal energy reads

(9) $$U_{ideal}=F_{ideal}+TS_{ideal}=\frac{3}{2}Nk_{B}T.$$

Amongst the main thermal statistical quantifiers, we have the disequilibrium *D* [12]

(10) $$D_{ideal}^{\left(N\right)}=\int d\Omega \;\rho_{ideal}^{2}\left(r,p\right).$$

*D* can also be obtained from the free energy as shown in Ref. [20].

One obtains

(11) $$D_{ideal}^{\left(N\right)}={\left(\frac{\lambda^{3}}{v}\right)}^{N}\;e^{-N}\;2^{-3N/2},$$

and, for the statistical complexity one has [12],

(12) $$C_{ideal}^{\left(N\right)}={\left(\frac{\lambda^{3}}{v}\right)}^{N}\;e^{-N}\;2^{-3N/2}\;\ln\left(e^{5N/2}\;{\left(\frac{\lambda^{3}}{v}\right)}^{-N}\right).$$

The complexity and *D* vanish for N→∞ and T→∞ [12,29]. It is useful to have a disequilibrium and complexity per particle:

(13) $$D_{ideal}=\left(\frac{\lambda^{3}}{v}\right)\;e^{-1}\;2^{-3/2},$$

(14) $$C_{ideal}=\left(\frac{\lambda^{3}}{v}\right)\;e^{-1}\;2^{-3/2}\;\ln\left(e^{5/2}\;{\left(\frac{\lambda^{3}}{v}\right)}^{-1}\right).$$

### 2.1. Van der Waals Scenario

Now we add to our former picture inter-particle interactions and face the Hamiltonian [1]

(15) $$H=H_{0}+\sum_{i<j}\;u_{ij},$$

with $u_{ij}=u(|r_{i}-r_{j}\left|\right)$ representing the interaction energy between molecules *i* and *j*, that depends only on the distance $r_{ij}=r_{j}-r_{i}$. One sums i,j over the N(N−1)/2 possible pairs [1].

Now, the associated canonical partition function (CPF) reads [1]

(16) $$Q_{N}\left(V,T\right)=Q_{N}^{\left(0\right)}\left(V,T\right)\;Z_{N}\left(V,T\right),$$

where $Q_{N}^{\left(0\right)}\left(V,T\right)$ is given by Equation (4). $Z_{N}\left(V,T\right)$ is an integral, denominated the configuration one (CI) [1,2]

(17) $$Z_{N}\left(V,T\right)=\frac{1}{V^{N}}\;\int d^{3N}r\;\exp\left(-\beta \sum_{i<j}\;u_{ij}\right).$$

For n=N/V≪1 the CI becomes [2]

(18) $$Z_{N}\left(V,T\right)\approx \exp\left(-\frac{NB_{2}\left(T\right)}{v}\right).$$

Here $B_{2}\left(T\right)$ is called the second virial coefficient [2]. One usually approximates the interaction in the form of a particular u(r)-form to be explained below (see Equation (22)) [2]. Then

(19) $$B_{2}\left(T\right)=\frac{1}{2}\;\int d^{3}r\;\left(1-\exp(-\beta u(r))\right).$$

The grand partition function is of the form [1]

(20) $$Z_{GC}=\sum_{N=0}^{\infty }\;z^{N}Q_{N}\left(V,T\right),$$

with $z=\exp\left(\mu_{vdW}/k_{B}T\right)$ called the fugacity. $\mu_{vdW}$ is the chemical potential [1]. Via Equation (16), we analytically obtain

(21) $$Z_{GC}=\exp\left(z\frac{V}{\lambda^{3}}e^{-B_{2}\left(T\right)/v}\right).$$

The essential van der Waals step is to take the particle–particle interaction as [12]

(22) $$u\left(r\right)=\left\{\begin{array}{cc} \infty & r<r_{o}\\ \exp(-\beta u(r))\approx 1-\beta u(r) & r>r_{o}, \end{array}\right.$$

where $r_{o}$ is the minimum possible distance between molecules [2]. One is led then, via Equation (19), to write

(23) $$B_{2}\left(T\right)=b-\beta a,$$

with $b=2\pi r_{0}^{3}/3$, with an average potential *a*

(24) $$a=2\pi \int_{r_{0}}^{\infty }\;dr\;r^{2}u\left(r\right).$$

### 2.2. The van der Waals (vdW) Thermal Quantifiers

Noting that $B_{2}=b-a/k_{B}T$ one has [11]

(25) $$F_{vdW}=F_{ideal}+\frac{Nk_{B}T}{v}\left(b-\frac{a}{k_{B}T}\right),$$

where $F_{ideal}$ is that of Equation (6) [1]. Now, following usual text book-recipes [1], from the relation

(26) $$N=z{\left(\frac{\partial \ln Z_{GC}}{\partial z}\right)}_{V,T},$$

we easily are led to a chemical potential of the form

(27) $$\mu_{vdW}=k_{B}T\ln\left(\frac{\lambda^{3}}{v}\;e^{B_{2}\left(T\right)/v}\right).$$

The entropy *S* is given by

(28) $$S_{vdW}=-{\left(\frac{\partial F_{vdW}}{\partial T}\right)}_{V,N},$$

that using Equation (25) becomes

(29) $$S_{vdW}=S_{ideal}-Nk_{B}\left(\frac{b}{v}\right),$$

where $S_{ideal}$ is gained from by Equation (8) [1]. The internal energy $U_{vdW}$ reads then

(30) $$U_{vdW}=F_{vdW}+TS_{vdW}=U_{ideal}-\frac{Na}{v},$$

where $U_{ideal}$ is given by Equation (9).

For the pressure $P_{vdW}$ one has

(31) $$P_{vdW}=-\frac{1}{N}{\left(\frac{\partial F_{vdW}}{\partial v}\right)}_{T,N},$$

so that using Equation (25) we encounter

(32) $$P_{vdW}=\frac{k_{B}T}{v}\left(1+\frac{b}{v}\right)-\frac{a}{v^{2}}.$$

For a dilute gas with v≫b (low density) one specializes this, in the case of the van der Waals gas, to [1]

(33) $$P_{vdW}=\frac{k_{B}T}{v-b}-\frac{a}{v^{2}}.$$

For the vdW distribution [11]

(34) $$\rho_{vdW}\left(r,p\right)=\exp\left(-\beta H\right)/Q_{N},$$

the disequilibrium *D* becomes

(35) $$D_{vdW}^{\left(N\right)}=\int d\Omega \;\rho_{vdW}^{2}\left(r,p\right),$$

that, per particle, reads

(36) $$D_{vdW}=D_{ideal}\;\exp\left(b/v\right),$$

where $D_{ideal}$ is found in (13). Thus, *C* per particle becomes

(37) $$C_{vdW}=D_{vdW}\left(S_{vdW}/k_{B}\right).$$

### 2.3. The vdW QC Passage and the Temperature Benchmark $T_{2}$

This is a central topic. One looks for it by following the prescriptions of Ref. [12]. We take (1) $\varepsilon =U_{vdW}/N$ equal to the energy per particle of the vdW gas and (2) $\mu =\mu_{vdW}$ as the chemical potential. Accordingly, the vdW classical regime is characterized by [12]

(38) $$\exp\left(\beta \mu_{vdW}\right)\ll \exp\left(\beta U_{vdW}\right).$$

Furthermore (see again Ref. [12]), the classical regime is characterized by [11]

(39) $$\left(\frac{\lambda^{3}}{v}\right)\;\exp\left(\frac{B_{2}\left(T\right)}{v}\right)\ll \exp\left(\beta U_{vdW}\right),$$

and thus

(40) $$\frac{\lambda^{3}}{v}\ll \exp\left(\frac{3}{2}-\frac{b}{v}\right),$$

so that, calling $T_{2}$ an indicator of the CQ passage temperature, the classical vdW regime is found at *T*’s such that [11]

(41) $$T\gg T_{2}=\frac{2\pi \hbar^{2}}{m\;k_{B}\;e\;v^{2/3}}\;\exp\left(\frac{2b}{3v}\right).$$

Note that the QC-passage’s indicative temperature $T_{2}$ is merely a theoretical benchmark, not an experimental quantity.

### 2.4. The Thermal Efficiency η

According to Ref. [28], if we regard the vdW constant *b* as a control parameter then

(42) $$\eta_{vdW}=-\frac{1}{k_{B}}\frac{\partial S_{vdW}/\partial b}{\partial F_{vdW}/\partial b}.$$

Via Equations (25) and (29), we come up with

(43) $$\eta_{vdW}=-\frac{1}{k_{B}T}.$$

For the classical–quantum passage’s indicative temperature $T_{2}$, Equation (43) yields

(44) $$\eta_{vdW}\left(T_{2}\right)=\eta_{2}=-\frac{me\;v^{2/3}\;e^{1-2b/3v}}{2\pi \hbar^{2}}.$$

In addition, we calculate, for the gas–liquid transition temperature $T_{c}$

(45) $$\eta_{vdW}\left(T_{c}\right)=\eta_{c}=-\frac{27b}{8a},$$

with $T_{c}=27k_{B}b/8a$ [11].

### 2.5. Boyle Temperature

The Boyle temperature can be defined as the point in the temperature range at which a real gas starts to behave like an ideal one. Define the reduced variables

(46) $$\hat{v}=\frac{v}{v_{c}},\;\;\;\;\hat{\tau }=\frac{\tau }{\tau_{c}},\;\;\;\;\hat{p}=\frac{p}{p_{c}},$$

with $\tau =k_{B}T$ and

(47) $$v_{c}=3b,\;\;\;\;\;\tau_{c}=\frac{8a}{27b},\;\;\;\;\;p_{c}=\frac{a}{27b^{2}},$$

the well-known critical points of the van dew Waals equation [6]. The so called Boyle temperature is the τ value [6]

(48) $${\hat{\tau }}_{B}=\frac{27}{8}\left(\frac{3\hat{v}-1}{3\hat{v}}\right).$$

**Figure 1:**
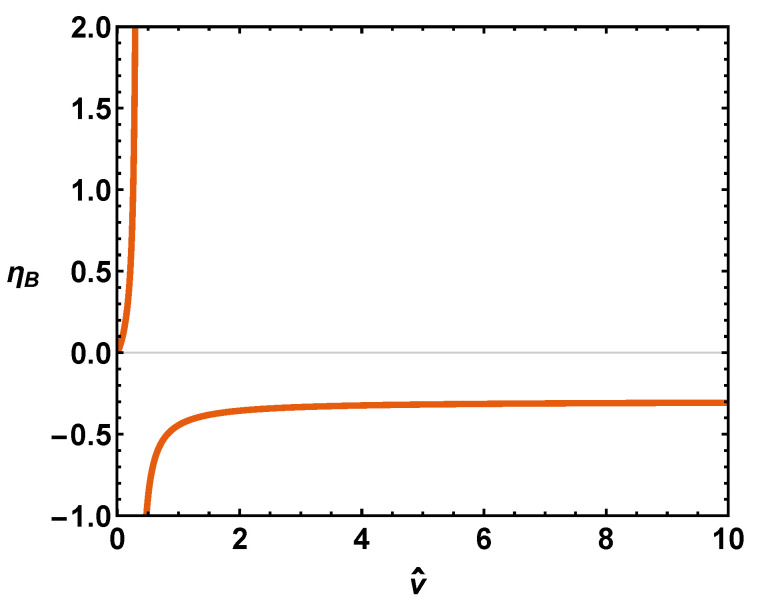
Thermal efficiency at the Boyle temperature versus $\hat{v}$. There is, of course, a singularity at close packing, as *b* can nor be diminished there.

The thermal efficiency here is

(49) $$\eta_{B}=\eta \left(\tau_{B}\right)=-\frac{8\hat{v}}{9(3\hat{v}-1)},$$

and diverges for $\hat{v}=1/3$, i.e., when v=b (close-packing). When $\hat{v}$ tends to infinity, one has $\eta_{B}=-1/{\hat{\tau }}_{B_{max}}=-8/27=-0.296$, with ${\hat{\tau }}_{B_{max}}=27/8$ being the limit of the Boyle temperature for large volumes ($\hat{v}\to \infty$) [6]. According to Ref. [6], the minimum value of ${\hat{v}}_{min}=1/3$. Thus, our $\eta_{vdW}<0$ always (see Figure 1). We have now collected all the necessary weaponry in order to tackle our job. We illustrate the usefulness of this quantifier in Figure 1. The thermal efficiency displays a singularity at close packing.

## 3. The Relation between our Two vdW-Significant Points for Five Noble Gases

We have two kinds of vdW-significant points:

- That which signals the gas–liquid phase transition. For this, we use the sub-index “c”, e.g., $T_{c}$.
- That which preannounces the classical–quantum passage. For this, we use the sub-index “2”, e.g., $T_{2}$.

**Figure 2:**
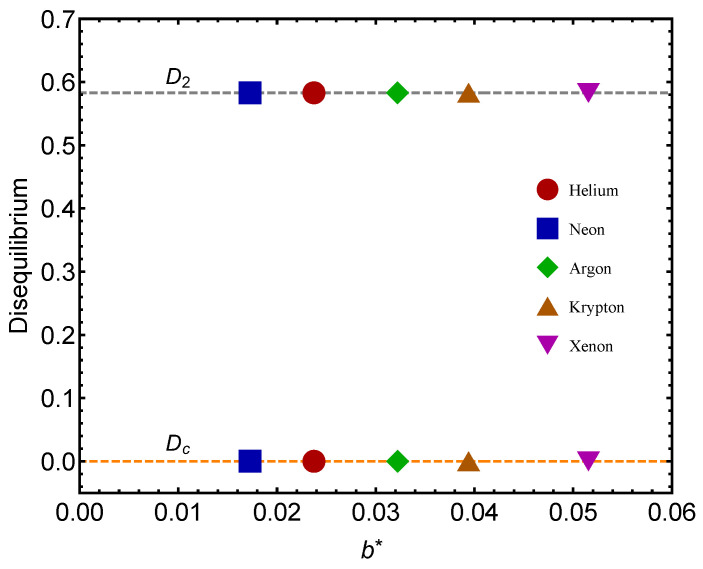
Disequilibrium *D* for each of the two benchmarks, $D_{c}$ for the GL and $D_{2}$ for the QC versus $b^{*}$. Icons correspond to fitted $b^{*}$-values. The lower values are ascribed to the GL passage. We take the molar volume v=1 L. We appreciate the notable fat that the QC passage is associated to a larger *D* than the GL one. Thus, the need to pass from the classical formalism to a quantum one seems to generate more statistical order than passing from the gas to the liquid phase. Note that, save for Helium, the difference QC–GL is a constant for the remaining gases. Note that $D_{c}$ vanishes in most instances. The lines are visual aids representing virtual trajectories as $b^{*}$ varies.

We deal with Helium, Neon, Argon, Krypton, and Xenon, vdW-parametrized by Johnston in Ref. [6]. The *b* parameter fitted for each gas is called $b^{*}=N_{A}b$, with $N_{A}$ the Avogadro constant [6]. We concoct a special icon for each of our noble gases (see Figure 2). In each of the following figures, gas-icons appearing in the upper portion of the graph correspond to the QC-values, while those depicted in the lower portion correspond to the GL-value. Additionally, the lines depicted in the graphs are visual aids.

Remember that the disequilibrium *D* signals statistical order. The larger *D*, the larger the order-degree of the concomitant system. In Figure 2, we consider the disequilibrium *D* for each of the two significant points: (1) gas–liquid (GL) $D_{c}$ and (2) quantum–classical (QC) $D_{2}$. We see that the *D*-values are different for the two benchmarks. The lower values in our graphs always correspond to $D_{c}$ (the GL phase). What is this fact telling us? That there is a larger ordering degree in the classical–quantum (CQ) passage than in the GL one, a novel fact discovered here. The ensuing differences for a given gas are much smaller for He than for the other gases. Let us emphasize that D=0.58 represents a high degree of statistical order, as $D_{maximum}=1$.

**Figure 3:**
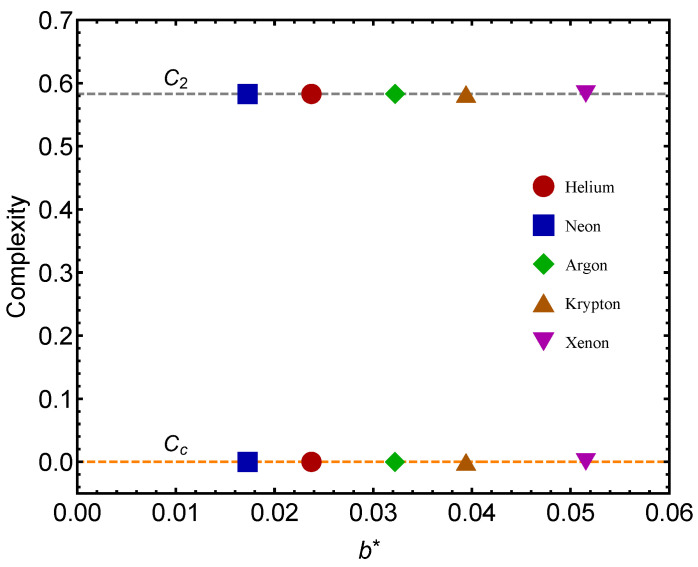
Complexity *C* for each of the two benchmarks (GL) and (QC) versus $b^{*}$. Icons correspond to fitted $b^{*}$-values. GL values are the lower ones. We take the molar volume v=1 L. We appreciate the notable fat that the QC passage is associated to a larger *C* than the GL one. Note that $C_{c}$ vanishes in most instances and that the complexity-difference QC–GL tends to be constant, save for He. The lines are visual aids representing virtual trajectories as $b^{*}$ varies.

Figure 3 is the counterpart of Figure 2 but for the complexity quantifier. The same rather surprising finding is here re-encountered. The vicinity of a quantum regime generates more statistical complexity than passing from the gas to the liquid phase, a novel fact discovered here. Note that the complexity-difference QC–GL is much smaller for He than for the remaining gases.

**Figure 4:**
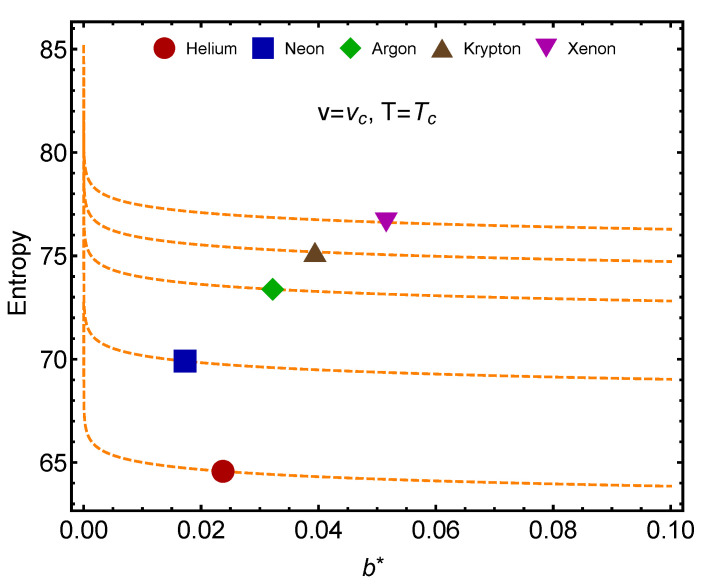
vdW entropy for $T=T_{c}$ and $v=v_{c}$ versus their proper $b^{*}$ for the noble gases. Helium’s entropy is not positive. This is so because the QC passage’s indicative temperature $T_{2}>T_{c}$. At $T_{c}$, He must therefore be treated in quantum fashion. Its liquid phase is strictly quantal and thus the classical vdW entropy does not make physical sense, becoming negative. The lines are visual aids representing virtual trajectories as $b^{*}$ varies.

In Figure 4, we deal with the vdW entropy *S* for the noble gases. *S* tends to grow with the molecule’s mass. Helium is a special case, as explained in the caption.

**Figure 5:**
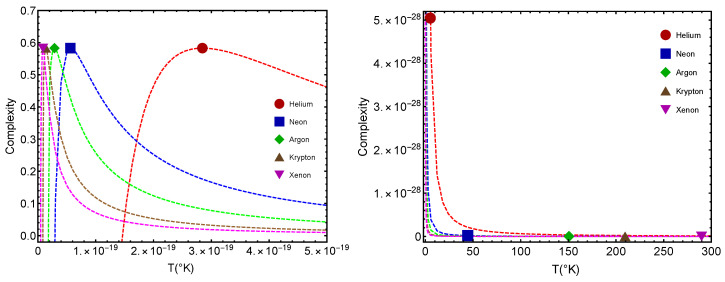
Complexity *C* versus *T* for five noble gases. The icons correspond to $T_{c}$ and $T_{2}$. Left, we find $C_{2}$ values. Right, the $C_{c}$ ones. Here we take v=1 L. We see that $C\left(T_{2}\right)>C\left(T_{c}\right)$. The lines are visual aids representing virtual trajectories as *T* varies. Note that $C_{2}$ is maximized at the Johnston-fitted [6] $b^{*}$-value.

In Figure 5 we inspect the complexities $C(T_{2})$ (larger values) and $C(T_{c})$ (smaller values) versus the temperature *T* for v=1 L. $T_{2}$ is the critical temperature for the QC passage, while $T_{c}$ is that for the GL one. Notably enough, for most of them, this difference between $C(T_{2})$ and $C(T_{c})$ is constant. Note that, consistently, $C\left(T_{2}\right)>C\left(T_{c}\right)$. This entails that complexity at the putative classical–quantum stage is larger than that at the gas–liquid one. Furthermore, the complexity is maximal at the benchmark $T_{2}$.

**Figure 6:**
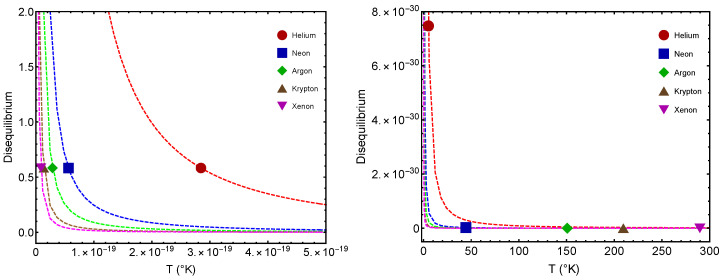
Disequilibrium *D* versus *T* for five noble gases. Left, we find $D_{2}$ values. Right, the $D_{c}$ ones. Here we take v=1 L. We note that $D\left(T_{2}\right)>D\left(T_{c}\right)$. The lines are visual aids representing virtual trajectories as *T* varies.

Figure 6 displays the disequilibrium-difference $d_{2c}=D\left(T_{2}\right)-D\left(T_{c}\right)$ for v=1 L. $T_{2}$ is the indicative temperature for the QC passage, while $T_{c}$ is that for the GL one. We consider the noble gases and find always $D\left(T_{2}\right)>D\left(T_{c}\right)$. Notably enough, for most of them (save for He), this difference $d_{2c}$ is constant. $d_{2c}$ is the “degree of statistical order-difference”, a constant here. The order–disorder disjunction is seen to display a characteristic vdW behavior. $D\left(T_{2}\right)>D\left(T_{c}\right)$ entails, let us insist, that the statistical order generated at the putative classical–quantum passage is larger than that at the gas–liquid one.

**Figure 7:**
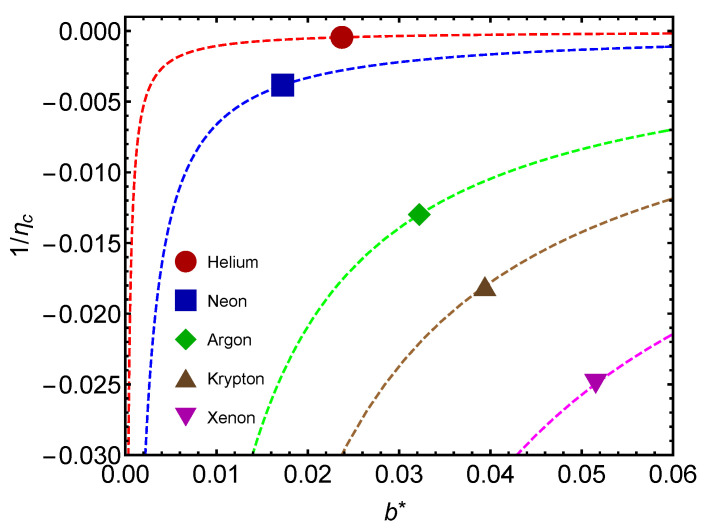
Noble gases’ inverse thermal efficiency versus their proper $b^{*}$ for $T=T_{c}$ and $v=v_{c}$ (Remember that Helium is a special case). The larger the atom’s mass, the more work is needed in modifying its $b^{*}$ value. The lines are visual aids representing virtual trajectories as $b^{*}$ varies.

We consider the thermal efficiency η in Figure 7. This quantity measures the work involved in changing the $b^{*}$-value. The larger the atom’s mass, the more work is performed by the atom in modifying its $b^{*}$-value.

In Figure 7, we encounter a major difference between (1) the GL and (2) the (putative) QC passages. This difference is related to the $b^{*}$-value. In the GL transition, the system needs to release energy to change its $b^{*}$-value. One might be reminded of the fact that when vapor condenses to a liquid, the vapor’s latent energy is released.

In the second passage (CQ) one must work on the atom to change that $b^{*}$-value. This is a typical quantum effect in the sense that the observer somehow becomes involved in dealing with quantum matters.

**Figure 8:**
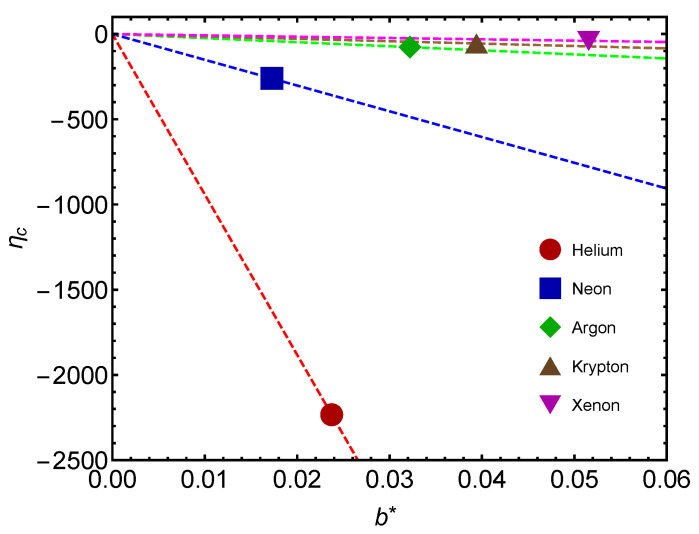
Critical thermal efficiency $\eta_{c}$ versus $b^{*}$ for noble gases. The icons correspond to the $b^{*}$ values fitted for noble gases. The lines are visual aids representing virtual trajectories as $b^{*}$ varies.

Another interesting plot is that of Figure 8. We depict $\eta_{c}$ versus $b^{*}$ for several gases. Values are negative, as expected from previous considerations. As mass augments, less work is involved in changing $b^{*}$.

## 4. Conclusions

In this work, we have performed a thermal statistical study of the (putative) classical–quantum frontier in a van der Waals scenario, with emphasis on the noble gases. For the purpose, we used rather novel thermal statistical quantifiers such as the disequilibrium, the statistical complexity, and the thermal efficiency. Fruitful insights have been thereby gained. The two benchmark temperatures are $T_{c}$ (GL) and $T_{2}$ (QC).

We discovered that there is a larger (statistical) ordering degree in the classical–quantum (CQ) passage than in the GL one. *This a novel fact discovered here*. The need to effect a “CQ change of appropriate describing-formalism” carries more order than the entirely classic vdW GL description. The same kind of comparison applies also to the statistical complexity. Furthermore, the differences $D\left(T_{2}\right)-D\left(T_{c}\right)$ and $C\left(T_{2}\right)-C\left(T_{c}\right)$ tend to be constant for most noble gases (He excluded).

In the GL transition, the system needs to release energy to change its $b^{*}$-value, while in the putative CQ passage we must do work on the atom to change that $b^{*}$ value, a notable difference between the two kinds of transformation. One might perhaps dare to interpret this work that we must do as that of Schrödinger–Heisenberg in creating quantum mechanics.

We emphasize the fact that the classical vdW-structure somehow anticipates, at low enough *T*, a jump in the statistical order-degree that we a posteriori associate with the quantum–classical frontier.

Our present classical van der Waals treatment is not capable to allow us to discuss out of equilibrium systems such as Josephson junction systems and many body systems [30,31,32].

## Data Availability

Everything that might be needed is here.
